# Holter Recordings at Initial Assessment for Long QT Syndrome: Relationship to Genotype Status and Cardiac Events

**DOI:** 10.3390/jcdd9050164

**Published:** 2022-05-23

**Authors:** Kathryn E. Waddell-Smith, Alexandra A. Chaptynova, Jian Li, Jackie R. Crawford, Halina Hinds, Jonathan R. Skinner

**Affiliations:** 1 Department of Cardiovascular Medicine, Flinders Medical Centre, Adelaide, SA 5042, Australia; kathryn.waddell-smith@sa.gov.au; 2 Department of Paediatrics, Child and Youth Health, University of Auckland, Auckland 1010, New Zealand; alex.chaptynova@gmail.com (A.A.C.); jian.li@middlemore.co.nz (J.L.); 3 The Cardiac Inherited Disease Group, New Zealand; jackiec@adhb.govt.nz; 4 Green Lane Paediatric and Congenital Cardiac Services, Starship Children’s Hospital, Auckland 1023, New Zealand; halinah@adhb.govt.nz; 5 Department of Child and Adolescent Health, University of Sydney, Camperdown, NSW 2006, Australia

**Keywords:** long QT syndrome, Holter monitor, risk stratification, diagnosis

## Abstract

Background: The relationship of Holter recordings of repolarization length to outcome in long QT syndrome (LQTS) is unknown. Methods: Holter recordings and initial 12 lead ECG QTc were related to outcome in 101 individuals with LQTS and 28 gene-negative relatives. Mean QTc (mQTc) and mean RTPc (R-wave to peak T-wave, mRTPc) using Bazett correction were measured, analyzing heart rates 40 to 120 bpm. Previously reported upper limit of normal (ULN) were: women and children (<15 years), mQTc 454, mRTPc 318 ms; men mQTc 446 ms, mRTPc 314 ms. Results: Measurements in LQTS patients were greatly prolonged; children and women mean mQTc 482 ms (range 406–558), mRTPc 351 ms (259–443); males > 15 years mQTc 469 ms (407–531), mRTPc 338 ms (288–388). Ten patients had cardiac arrest (CA), and 24 had arrhythmic syncope before or after the Holter. Holter values were more closely related to genotype status and symptoms than 12 lead QTc, e.g., sensitivity/specificity for genotype positive status, mRTPc > ULN (89%/86%); CA, mRTPc > 30 ms over ULN (48%/100%). Of 34 symptomatic (CA/syncope) patients, only 9 (26%) had 12 lead QTc > 500 ms, whereas 33/34 (94%) had an mRTPc or mQTc above ULN. In 10 with CA, all Holter measurements were > 15 ms above ULN, but only two had 12 lead QTc > 500 m. Conclusions: Holter average repolarization length, particularly mRTPc, reflects definite LQTS status and clinical risk better than the initial 12 lead QTc. Values below ULN indicate both a low risk of having LQTS and a low risk of cardiac events in the small percentage that do.

## 1. Introduction

Long QT syndrome (LQTS) predisposes to malignant ventricular arrhythmias causing cardiac arrest, predominantly in the young [1]. Diagnosis can be challenging due to the variation in clinical expression [2], with significant overlap in QT intervals between affected and unaffected individuals [3,4] and difficulties in measuring QT intervals [5]. Risk stratification and allocation of appropriate therapy currently relies on either the manifestation of symptoms, which are often life threatening, or a corrected QT interval (QTc) exceeding 500 ms [1], which is frequently unrecognized by the majority of physicians [5]. Before these high-risk features manifest, the young patient is exposed to a higher risk; unnecessary had they been identified at initial assessment. We wondered if Holter measurements might help identify a high- or low-risk group at the outset, on the first review at clinic, when it is not even certain whether a family member is a gene carrier or not.

The use of Holter recordings in the assessment of LQTS has previously been studied with variable results. Some have demonstrated a higher proportion of pathological ventricular arrhythmias [6], and others have not [7], although a longer QTc in LQTS subjects is commonly seen [3,7,8]. Some of these techniques appear to be complex, and repeatability has not been established [8,9]. Thus, they may not be widely applicable.

We recently published normative values of mean repolarization length using the Bazett heart-rate correction formula restricting measurements to heart rates between 40 and 120 beats per minute (BPM) [10]. The resultant measurements were found to be highly repeatable. The measurement of the R wave to peak T wave (RTPc) (as opposed to the Q wave to the end of the T wave, QTc) was the most repeatable.

We aimed to determine if these mean measurements of repolarization length might be superior to the 12-lead ECG in differentiating those with definite LQTS from genotype-negative family members and in assessing risk of arrhythmic syncope and cardiac arrest.

## 2. Materials and Methods

This study was approved by the Health and Disability Ethics Committee New Zealand and the local area health board: number: 16/NTB/86.

### 2.1. Study Population

Subjects being assessed for possible LQTS in a regional clinic [11] underwent Holter recording analysis in addition to usual personal and family history and 12-lead ECG analysis.

Holter recordings were obtained to review for arrhythmias and T-wave alternans prior to beta-blocker therapy in all but 35 and were stored for subsequent assessment of repolarization length. Subjects were excluded if they had other congenital or acquired cardiac conditions or were known to be taking QT-prolonging medications. Genotype status was subsequently ascertained [11]. 

ECGs were at least 72 h post-cardiac arrest and were performed without known electrolyte disturbances or in the presence of QT-prolonging medications. For the purposes of this study, we report the first ECG in the medical record fitting these criteria.

All patients or their guardians provided written informed consent to participate in the New Zealand Cardiac Inherited Diseases Registry and have their anonymized clinical and genetic data used for research into their condition (as previously described) [11].

### 2.2. Data Collection

#### 2.2.1. Patient Information

Demographic data and personal histories were collected from medical records, most of which were stored prospectively as part of the registry. Cardiac arrest was defined as sudden cardiac death or resuscitated cardiac death (requiring bystander CPR or DC cardioversion) with or without documented ventricular tachycardia. Syncope was defined as an abrupt, transient loss of consciousness with spontaneous recovery. Clinically apparent vasovagal syncope was excluded.

#### 2.2.2. ECG Analysis

The first three authors (KWS, AC, JL) performed the 12-lead ECG analysis; each was blinded to patient identity, genotype, and clinical outcome. The QT interval was measured from the beginning of the QRS complex to the end of the T-wave, as defined by the “tangent technique”, where the tangent of the steepest slope of the second limb of the T-wave crosses the isoelectric line [12]. The longest measurement of lead II or V5 was taken and was compared with age and sex-based reference ranges and classified as prolonged (children 1–15 years old: ≥460 ms, men: ≥450 ms, and women: ≥470 ms) [13] and if ≥500 ms [1].

#### 2.2.3. Holter Recordings and Analyses

All study subjects underwent a 3-lead 24 h Holter recording, as previously described [10]. Briefly, the Holter recordings began in the year 2000 at initial family assessment using LifeCard CF monitors (Ecomed, Seven Hills, NSW 2147, Australia) and Del Mar Impressario software (Washington, DC, USA). QTc was calculated for heart rates between 40–120 beats per minute only, by an experienced cardiac technician (HH). This commercially available method uses semi-automated measurement (Laguna algorithm) [14] with caliper placement and periodic adjustment by the technician. The end of the T wave is determined by the zero-crossing point with the Holter, tending to give longer measurements than those made using the tangent technique on the 12 lead ECG.

R-wave to peak T-wave (RTP) was also recorded and corrected in the same way for heart rate (RTPc). 

Measurements in children (<15) were remarkably similar to adult females and were grouped together. Upper limits of normal (ULN) were defined as 2SD above the mean and were as follows: males > 15 years, mean QTc 446 ms, mean RTPc 314 ms; children and females > 15 years, mean QTc 454 ms, mean RTPc 319 ms.

The median length of recording for beats analyzed within the heart rate range was 18.2 h, maximum 23 h, minimum 4 h (in an infant).

#### 2.2.4. Statistical Analyses

Assumptions of the t-test were tested, and all data were analyzed by unpaired parametric and non-parametric t-tests as appropriate. Continuous variables are expressed as mean ± standard deviation or median and interquartile range (IQR). Chi-squared tests were used for binary variables. Statistical analyses were performed using GraphPad Prism version 6.0e for Mac, GraphPad Software, La Jolla, CS, USA, www.graphpad.com, accessed on 12 February 2019.

Mean QTc, and RTPc intervals were compared using one-way ANOVA. These statistics were performed using SAS statistical package, version 8, SAS Institute, Cary, NC, USA. In all cases, two-tailed *p* < 0.05 were considered statistically significant.

## 3. Results

### 3.1. Study Population

One hundred twenty-nine subjects who were being assessed for possible LQTS in a regional clinic were included in the study population.

Six probands either did not have genetic testing performed or had an uninformative result; all six had a Schwartz score ≥4. The LQTS group (101 subjects) comprised 95 gene-positive individuals, including 39 probands and 56 gene-positive relatives, and 6 gene-unknown probands. The age range of the LQTS subjects was 3 months to 67 years, comprising 53 females and 48 males; 53 were under 15 years of age. The 28 individuals who were gene negative for the disease-causing variant identified within the family formed the “gene negative” cohort. Raw data are available for review in Supplementary Table S1.

### 3.2. ECG Findings

There has been a median of 8.6 years since the initial ECG (or Holter monitor if date of first ECG unavailable) (range 0 days–21 years); 1000 patient-years.

We examined the utility of this investigation in (1) diagnosis and (2) risk stratification of those with possible LQTS.

### 3.3. Genotype Status

Of the 95 genotype positive individuals, 71 had a *KCNQ1* mutation (median age 13, range 0 months–67 years, IQR 6–35, 49% male), 23 had a *KCNH2* mutation (median age 8, range 3 months–49 years, IQR 4–16, 43% male), and 1 had Jervell and Lange-Nielsen Syndrome (10-year-old female). Six probands with uninformative tests, but Schwartz score > 4 had a median age of 13 (range 10–16, IQR 11–15), and 50% were male. Twenty-eight genotype-negative relatives have remained asymptomatic during follow-up.

### 3.4. Symptomatic Status

In the LQTS group of 101 subjects, 10 individuals suffered cardiac arrest, 24 experienced arrhythmogenic syncope as their most severe symptom, and 67 never had a symptom. 

Cardiac arrest occurred prior to the Holter recording in 8/10 cases and in two during follow-up, with a minimum of one week between the event and recording. The age at cardiac arrest occurred at a median of 15 years (range 6 months–41 years).

### 3.5. Diagnosis of LQTS Using Holter mQTc and mRTPc

**Figure 1:**
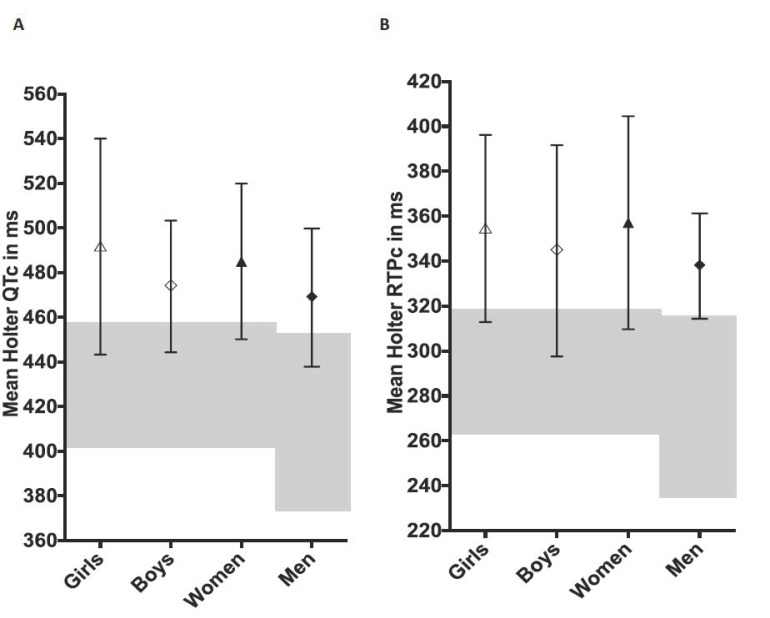
Holter-derived mean QTc (Panel (**A**)) and RTPc values (Panel (**B**)) from 101 subjects with long QT syndrome, values are mean ± SD (ms, milliseconds). “Girls”—females < 15 years and “Boys”—males < 15 years. Normative ranges are shown in grey shading. The central motif indicates the mean value.

The LQTS cohort had significantly longer Holter repolarization length results than the relevant normative cohort, see Table 1 and Table 2 and Figure 1. 

For the purposes of this study and to permit studies of specificity and sensitivity, we defined the upper limits of normal (ULN) for each measurement as that equaling two standard deviations above the normative mean from our previous report. (We recognize that some normative values lie above this and show the highest normative values in Table 2). Sensitivity and specificity for the confirmed genetic diagnosis of LQTS based on values exceeding the age and sex normative values are shown in Table 3.

There was no significant difference in measurements between the groups.

### 3.6. Predicting Risk of Syncope and Cardiac Arrest Using Holter Measurements and the Initial 12 Lead ECG

We compared the results of the cardiac arrest cohort and those with arrhythmic syncope with the remainder of the LQTS group and the gene-negative cohort. Holter measurements were higher in those who had a cardiac arrest compared to those who did not. The average (SD) mQTc was 495 ms (23) in those with cardiac arrest compared to 470 ms (SD 39) in those without (*p* = 0.05). Results were similar for mRTPc measurements (average mRTP 365 ms (SD 20) versus 331 ms (SD 37), *p* = 0.049).

**Figure 2:**
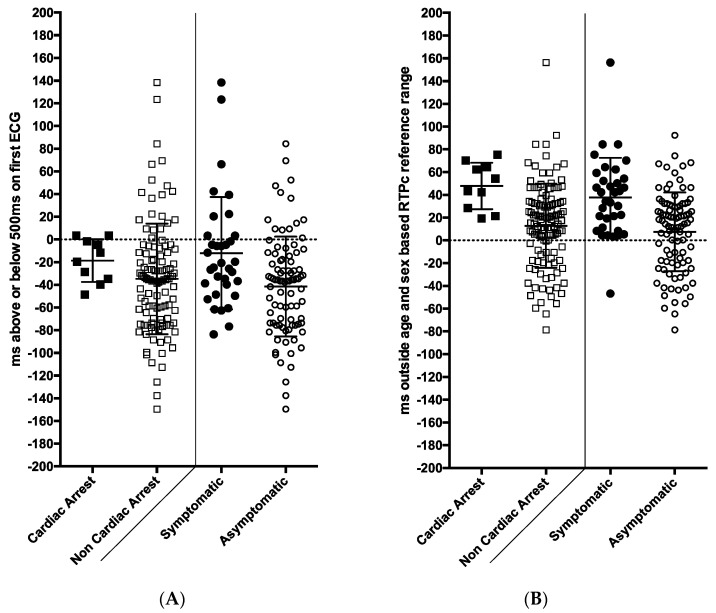
The association of ECG and Holter recording measurements with symptoms and cardiac arrest among families presenting for assessment with long QT syndrome (101 patients with long QT syndrome and 28 gene negative family members). (**A**) Panel shows QTc values from the initial 12 lead ECG expressed as milliseconds above the age/sex-based reference values. Only 2/10 patients who had cardiac arrest (left side) and 7/24 symptomatic patients (right side) had a QTc exceeding 500 ms on their first ECG. (**B**) This panel shows Holter-derived mean RTPc values (R wave to peak T wave corrected for heart rate) expressed as milliseconds above the age/sex-based reference range. Results from 10 cardiac arrest survivors are shown on the left versus the rest, and on the right the symptomatic versus asymptomatic subjects are shown. Only one symptomatic patient had a value in the normal range. ms = milliseconds. Black circle—LQTS with symptoms, black square—LQTS with cardiac arrest, white circle—all subjects (gene positive and negative) without symptoms, white square—all subjects (gene positive and negative) without cardiac arrest.

However, these measurements make no allowance for age and gender differences. When each mQTc or mRTPc value was then expressed as a millisecond deviation above or below the age/sex-based upper limit of normal (ULN), the groups became more separated. Figure 2 demonstrates the distribution of values from the initial 12 lead ECG and for the Holter-derived mRTPc. It is striking that the lowest mRTPc value in a subject with cardiac arrest was 19 ms above the ULN.

We thus elected to use a cut-off of 15 ms above the ULN for age/sex-based Holter mRTPc recordings to calculate sensitivity and specificity for the prediction of genotype status, syncope, and cardiac arrest.

A similar process was then conducted for the initial 12 lead QTc using a QTc above upper limits of normal for age and gender, and also for a QTc above 500 ms. The results are summarized in Table 3.

We then compared two groups: symptomatic LQTS individuals (who experienced either cardiac arrest or syncope) against asymptomatic individuals (comprising both asymptomatic LQTS individuals and the gene-negative cohort). This was done to represent the clinical scenario of a family member in the clinic with unknown genotype status. Symptomatic LQTS individuals had significantly longer Holter mQTc than the asymptomatic cohort; mean 491 ms (SD 41) versus 465 ms (SD 36), respectively, *p* = 0.008. Similarly, they had longer mRTPc values; mean 356 ms (SD 35) versus mean 326 ms (SD 35), *p* < 0.0001.

When the data were expressed as milliseconds above the age/sex-based reference range, the significant differences between the symptomatic and asymptomatic groups remained (mean QTc *p* = 0.0005 and RTPc *p* < 0.0001), see Figure 2. *Only one symptomatic patient (cardiac arrest or syncope) had an mRTPc in the normal range*.

**Figure 3:**
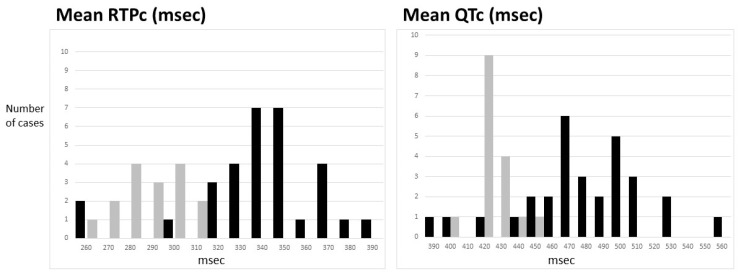
Holter-derived mean QTc and RTPc values are shown from those less than 7 years of age. Normative controls (*n* = 16) are shown in grey, and those with long QT syndrome (*n* = 31) are shown in black. Values were rounded up or down to the nearest 10 ms.

Holter values in children less than 7 years may be of particular significance given the difficulty in obtaining exercise tests in this group. Those with LQTS are compared against normative controls in Figure 3. The results are similar to the entire group; mRTPc ≥ 320 ms or mQTc > 460 ms was only seen in those with LQTS. However, there was an overlap in genotype-positive individuals into the normal range; 3 of 31 (10%) for mRTPc and 6 of 31 (19%) for mQTc.

### 3.7. Comparison of Holter Measurements with 12-Lead ECG Measurements

A QTc less than 500 ms on the initial 12 lead ECG was seen in 17/24 (71%) patients with syncope and 8/10 (80%) patients with cardiac arrest. In comparison, a Holter mRTPc or mQTc > 15 ms above the ULN was seen in all ten patients with cardiac arrest (i.e., a negative predictive value of 100%).

Only one patient of 24 with syncope had a Holter mQTc and mRTPc below the age/sex-based ULN.

## 4. Discussion

This exploratory study of Holter recordings in 101 individuals with long QT syndrome, and 28 family gene negative individuals, demonstrates that the Holter measurements of the average length of repolarization are superior in diagnosing LQTS disease status than the QTc from the initial 12 lead ECG. Among subjects with definite LQTS, 31% had a normal QTc on the initial 12 lead ECG, but only 11% had a normal Holter-derived mRTPc.

This analysis does not account for qualitative assessment of T wave morphology on the 12 lead ECG—which can alert specialists, in particular, to genotype-positive status, even when the QTc is within the normal range. Nevertheless, the findings are striking. Other researchers have had similar findings. Follansbee et al., in a study of 39 patients with long QT syndrome, found a cut-off mean QTc of over 461 ms was 99% specific (and 79% sensitive) for genotype-positive status versus controls [15]. We found that it was only by dealing with adult males separately, that we could get predictions this good in our larger and more diverse cohort (e.g., 89% specificity and 86% specificity for an mRTPc value over the upper limit of normal). 

Prolonged Holter values also had a superior association with syncope and cardiac arrest than QTc from the 12 lead ECG. A QTc greater than 500 ms on a 12 lead ECG is a well-established indicator of risk of cardiac events in long QT syndrome. However, 17 of 24 who experienced syncope (71%), and 8 of 10 patients who experienced cardiac arrest (80%), had a QTc less than 500 ms on their initial ECG. Yet, 33 of 34 symptomatic patients had an mRTPc or mQTc above the upper limit of normal, and all ten patients who suffered a cardiac arrest had an mRTPc and mQTc > 15 ms over the upper limit of normal. No other study has reported this finding. However, it is not the first to suggest that Holter recordings of QTc may be linked to adverse outcomes. Page et al. [16] showed that failure of the QT to adapt to changes in heart rate was associated with a higher risk of symptoms, building on the concept of the “QT clock”, where different genotypes demonstrate phenotype-specific QT rate behavior [9]. Holter measurements of QT interval and minor T wave alternans are also linked to adverse outcomes in adult patients with heart failure [17,18], even when limited to only 30 min recordings [19]. 

This study finds that normative Holter mRTPc and mQTc measurements are uncommon in long QT syndrome (10–15%), and longer values are more commonly associated with syncope or cardiac arrest (occurring before or after the test). The specificity of mRTPc for genotype status and adverse event was slightly better than mQTc. This may be a technical issue—the automated detection of the peak component of the T wave is more reliable than the detection of the end of the T wave or a phenomenon affecting late repolarization in families who have long QT syndrome, even among those who do not carry the main genetic defect.

We have previously shown that these Holter measurements of mean QTc and RTPc were much more repeatable than QTc measurements from the 12 lead ECG [5]. This is another reason why this Holter technique may be a useful additional clinical tool for assessment of risk in those having, or suspected to have, long QT syndrome.

Mean QTc and RTPc in men was lower in normal subjects [20]. In the practical sense, it means that the normative range is lower in adult men than in all other groups and that results must be considered against the upper limits of normal by age and sex. We suspect that the normative range in men may, in fact, be lower than we report because the sample size was small. Although the highest value seen was 303 ms, 2SD above the mean was 315 ms, due to the small sample size. If this is the case, then sensitivity and specificity of the test are likely to improve with a larger normative cohort.

It is known that 12 lead ECG has significant limitations in the diagnosis of LQTS due to overlap with the normal population [2,3,4] and difficulties in QT interval measurement [5]. In terms of predicting the disease status, the poor sensitivity of the QTc recorded on 12 lead ECG at the initial assessment (69%) contrasts with the high sensitivity of the Holter RTPc measurements (89% Table 3). As with any biological variable, there is a trade-off between sensitivity and specificity of diagnosis. An initial 12 lead QTc > 500 ms was 100% specific for definite LQTS disease status, but less than a quarter would have been detected (23% sensitive). A Holter mRTPc value 30 ms above the upper limit of normal similarly was 100% specific, but had the improved sensitivity of 48%. In other words, the degree of overlap between those with disease and those without is less for the Holter measurements than for the 12 lead ECG measurements. These Holter measurements may therefore be a useful adjunct to the standing test and exercise provocation in determining phenotype and has the additional advantage of ease of use in young children and infants. 

The ultimate goal in LQTS management is preventing sudden cardiac death. It is already known that prior syncope or a QTc > 500 ms on the worst ECG recorded during follow-up indicates a high-risk individual [21], but in many patients, no clear risk factors are present on initial consultation, and accurate risk assessment is thus not possible. In the present study, although six of the ten patients suffering cardiac arrest eventually developed QT prolongation over 500 ms during their follow-up, it took up to 14.5 years to do so. In contrast, no cardiac arrest survivor had a Holter mRTPc or mQTc less than 15 ms above the ULN, giving a 100% negative predictive value for the test. In other words, values within the normal and borderline range may identify those where we may be more confident of a benign course. This would be analogous to having repeated normal 12 lead ECGs during follow-up, but with the information available right at the start. 

The study has a number of weaknesses and limitations. The software we used, which was first used over 20 years ago, did not allow us to use different heart rate correction formulae. We took a pragmatic approach to heart rate limitation at the outset (40–120 bpm). We cannot undo that and reanalyze all heart rates, although this would be interesting for future studies. Encouragingly, recent work has suggested that the Bazett formula is, in fact, adequate even for young children and infants with fast heart rates [22]. 

We do not know yet how these results will transfer to other reporting systems. We have not been able to study diurnal variation [9]. We also were not able to analyze changes in T-wave morphology, a science that has considerable promise. We elected to amalgamate patients with LQTS types 1 and 2, and several gene unknown patients to enable a sufficient sample size of patients who suffered cardiac arrest. We have no subjects with the less common forms of LQTS in the study, such as LQTS types 3, 5, and 6, and we do expect the different genotypes to behave differently [16]. Given the promising results of this study, it would be useful in the future to investigate these measurements according to a specific genotype. Further, we have not systematically compared the results to those of exercise tests or standing provocation [23,24,25,26]. This was not possible because this data was collated over 17 years, the value of the tests was not known at the start of the study, and almost a third of the cohort were under 7 years of age. It will also be interesting to see if heart rate restriction (40–120) is necessary or not, and whether this or other factors explain why some other researchers have not found such a strong link between Holter measurements and outcome [27].

## 5. Conclusions

This study introduces a new Holter technique to assess individuals with possible LQTS, and to assess their risk of symptoms and cardiac arrest. The most reproducible and potentially clinically useful measurement of repolarization length was the mean RTPc, the R wave to the peak of the T wave—related to normative age and sex-specific ranges. Upfront assessment of individuals potentially affected by LQTS with this simple and reproducible technique potentially allows high sensitivity and specificity of LQTS diagnosis and, to date, a 100% negative predictive value for the occurrence of cardiac arrest for values in or marginally above the normal range. If this work is supported by studies in other cohorts, it may become a useful adjuvant to the 12 lead ECG, the standing ECG, and exercise testing in determining phenotype, and may add to clinical history and serial 12 lead ECGs in determining the level of risk in long QT syndrome.

Holter-derived mean QTc values over 460 ms (or mean RTPc > 325 ms) in women and children, or over 450 ms (or mean RTPc > 320 ms) in men, are uncommon in the healthy population. In the initial assessment for possible long QT syndrome, values below these limits indicate both a low risk of having the condition and a low risk of cardiac events in the small percentage that do.

## Figures and Tables

**Table 1:** Initial 12 lead ECG QTc and Holter mQTc and mRTPc values for LQTS subjects and gene negative relatives (Mean (SD)).

		All < 15 Years	All < 15 Years and Female ≥ 15 Years	Female ≥ 15 Years	Male ≥ 15 Years
28 Family gene-negative subjects	Initial ECG QTc (ms)	420 (30)	418 (30)	413 (31)	430 (13)
	Mean Holter mQTc (ms)	436 (30)	436 (27)	435 (12)	436 (22)
	Mean Holter mRTPc (ms)	291 (26)	292 (25)	291 (10)	285 (15)
101 Subjects with LQTS *	Initial ECG QTc (ms)	477 (42)	481 (43)	492 (45)	461 (43)
	Mean Holter mQTc (ms)	481 (39)	482 (38)	485 (35)	469 (31)
	Mean Holter mRTPc (ms)	349 (45)	351 (46)	357 (47)	338 (25)

ms, milliseconds; mQTc, mean QTC measurement over 24 h; mRTPc, mean RTPc (R wave to peak T wave) measurement over 24 h. * 95 genotype-positive LQTS and 6 probands with unequivocal gene-unknown LQTS. All values were significantly higher in the LQTS subjects, *p* < 0.01.

**Table 2:** Previously published normative Holter recording measurements [10]. The normative reference range is shown in bold in the third column. Noting that some normative values are higher than this, the highest values seen are in the fourth column.

	Mean Value (SD) ms	Lower and Upper * Limits (Mean − 2SD and Mean + 2SD) ms	Highest Normative Mean Value Recorded, ms
**mQTc** all females and males < 15 years	424 (15)	**394–454**	467
**mQTc** males ≥ 15 years	408 (19)	**370–446**	436
**mRTPc** all females and males < 15 years	291 (14)	**263–319**	325
**mRTPc** males ≥ 15 years	274 (20)	**234–314**	303

ms, milliseconds; mQTc, mean QTC measurement over 24 h; mRTPc, mean RTPc (R wave to peak T wave) measurement over 24 h. * The upper limit of normal (ULN) was defined in the present study as 2SD above the mean for that group.

**Table 3:** Comparison of the sensitivity and specificity of initial 12-lead ECG QTc measurements and Holter-derived mQTc and mRTPc for the diagnosis and risk stratification of LQTS among 101 subjects with definite LQTS and 28 family gene-negative individuals.

		**Sensitivity (%)**	**Specificity (%)**
**LQTS disease status**			
First ECG	QTc prolonged QTc > 500 ms	69 23	92 100
Holter mQTc > ULN		85	75
Holter mRTPc > ULN		89	86
Holter mRTPc > 30 ms above ULN		48	100
**Risk of Cardiac Symptoms**			
First ECG	QTc prolonged QTc > 500 ms	74 24	26 85
Holter mQTc > ULN		94	36
Holter mQTc > 30 ms above ULN		47	69
Holter mRTPc > 30 ms above ULN		74	51
**Risk of Cardiac Arrest**			
First ECG	QTc prolonged QTc > 500 ms	80 20	46 82
Holter mQTc > ULN		90	29
Holter mQTc > 30 ms above ULN		70	66
Holter mRTPc > 30 ms above ULN		100	48

QTc on 12 lead ECG is defined as prolonged by age and gender; aged 1–15 > 460 ms, males > 15 years > 450 ms, females > 15 years > 470 ms. (ULN for mQTc and mRTPc is shown in Table 1).

## Data Availability

Raw data are not provided with this study, generalized publication is not usually permitted by the registry. However, de-identified tabulated results can be requested from the Corresponding Author by those with a legitimate research interest in the area.

## Supplementary Materials

The following supporting information can be downloaded at: https://www.mdpi.com/article/10.3390/jcdd9050164/s1, Table S1: Raw data for ECG Holter LQT outcome study.
