# Supplementary material for: Holter Recordings at Initial Assessment for Long QT Syndrome: Relationship to Genotype Status and Cardiac Events
Source: J Cardiovasc Dev Dis. 2022 May 23;9(5):164. doi: 10.3390/jcdd9050164 (PMC9147587; doi:10.3390/jcdd9050164)
Supplement: Supplementary file 1 [file jcdd-09-00164-s001.zip › jcdd-1673827-supplementary.pdf]

Table S1. GENE NEGATIVE SEX AGE AT HOLTHOLTER mQT HOLTER mQT mQTc ABOVE HOLTER mRT HOLTER mRT mRTPc ABOV FIRST QTc PROLONGED QTc 500 FIRST ECG ABFIRST ECG ABOVE 500ms age at 1/7/16.

**1**	**F**	**4**	**410**	**454**	**-44**	**286**	**319**	**-33**	**387**	**460**	**500**	**-73**	**-113**	**16.2849315**
2	F	6	422	454	-32	299	319	-20	430	460	500	-30	-70	6.19178082
3	F	6	486	454	32	319	319	0	411	460	500	-49	-89	22.7150685
4	F	8	450	454	-4	324	319	5	478	460	500	18	-22	24.7369863
5	F	9	419	454	-35	277	319	-42	420	460	500	-40	-80	23.5616438
6	F	10	445	454	-9	240	319	-79	426	460	500	-34	-74	26.4520548
7	F	11	411	454	-43	269	319	-50	424	460	500	-36	-76	16.860274
8	F	13	434	454	-20	300	319	-19	398	460	500	-62	-102	15.7835616
9	F	15	428	454	-26	306	319	-13	422	460	500	-38	-78	25.2712329
10	F	16	432	454	-22	276	319	-43	425	470	500	-45	-75	30.0136986
11	F	19	430	454	-24	289	319	-30	362	470	500	-108	-138	26.6410959
12	F	21	428	454	-26	293	319	-26	456	470	500	-14	-44	34.3178082
13	F	37	457	454	3	303	319	-16	411	470	500	-59	-89	53.5452055
14	F	50	429	454	-25	294	319	-25	399	470	500	-71	-101	63.4027397
15	M	2	412	454	-42	263	319	-56	-	460	500	-	-	13.9780822
16	M	2	406	454	-48	270	319	-49	409	460	500	-51	-91	18.6328767
17	M	4	488	454	34	337	319	18	-	460	500	-<br>-		20.5808219
18	M	5	432	454	-22	290	319	-29	426	460	500	-34	-74	19.1808219
19	M	5	400	454	-54	254	319	-65	435	460	500	-25	-65	11.1726027
20	M	6	407	454	-47	285	319	-34	350	460	500	-110	-150	17.6246575
21	M	7	462	454	8	299	319	-20	426	460	500	-34	-74	23.490411
22	M	9	460	454	6	310	319	-9	465	460	500	5	-35	25.660274
23	M	12	498	454	44	348	319	29	441	460	500	-19	-59	28.460274
24	M	13	416	454	-38	281	319	-38	391	460	500	-69	-109	27.7917808
25	M	45	418	446	-28	270	314	-44	419	450	500	-31	-81	45.0630137
26	M	48	433	446	-13	276	314	-38	418	450	500	-32	-82	61.9068493
27	M	51	468	446	22	303	314	-11	441	450	500	-9	-59	50.7150685
28	M	54	424	446	-22	289	314	-25	440	450	500	-10	-60	53.7561644
